# Yi-Zhi-Fang-Dai Formula Protects against A*β*_1–42 _ Oligomer Induced Cell Damage via Increasing Hsp70 and Grp78 Expression in SH-SY5Y Cells

**DOI:** 10.1155/2016/8591656

**Published:** 2016-10-17

**Authors:** Lumei Liu, Wenbin Wan, Wenjing Chen, Yuanjin Chan, Qi Shen, Yaming Li

**Affiliations:** ^1^Geriatrics Department of Chinese Medicine, Huadong Hospital, Fudan University, Shanghai, China; ^2^Department of Integrated Chinese and Western Medicine, Huashan Hospital, Fudan University, Shanghai, China; ^3^Department of Neurology, Zhongshan Hospital, Fudan University, Shanghai, China; ^4^Shanghai Jiao Tong University School of Pharmacy, Shanghai, China

## Abstract

Yi-Zhi-Fang-Dai formula (YZFDF) is an experiential prescription used to cure dementia cases like Alzheimer's disease (AD). In this study, the main effective compounds of YZFDF have been identified from this formula, and the neuroprotective effect against A*β*_1–42_ oligomer of YZFDF has been tested in SH-SY5Y cells. Our results showed that YZFDF could increase cell viability and could attenuate endothelial reticula- (ER-) mediated apoptosis. Evidence indicated that protein folding and endothelial reticula stress (ERS) played an important role in the AD pathological mechanism. We further explored the expression of Hsp70, an important molecular chaperon facilitating the folding of other proteins, and Grp78, the marker protein of ERS in SH-SY5Y cells. Data told us that YZFDF pretreatment could influence the mRNA and protein expression of these two proteins. At last, we also found that YZFDF pretreatment could activate Akt in SH-SY5Y cells. All these above indicate that YZFDF could be a potent therapeutic candidate for AD treatment.

## 1. Introduction

Alzheimer's disease (AD) is an age-related neurodegeneration disease which destroys cognitive function and eventually leads to death. Murray and colleagues [1] reported that AD had increased more in rank (from 32 to 9) of years lost to life because of premature mortality compared with any other major disease from 1990 to 2010. In the same year, a large-scale systematic analysis of the epidemiology of AD in China [2] showed that the incidence of dementia was 9.87 cases per 1000 person-years, and that of AD was 6.25 cases per 1000 person-years from 1990 to 2010. As there exists little treatment which can cure or slow down the progression of the disease, the socioeconomic impact of AD is growing steadily as the population is aging. The neuropathological features of AD are the formation of extracellular amyloid plaques (AP) composed of *β*-amyloid (A*β*) peptides [3, 4], the intracellular neurofibrillary tangles built by hyperphosphorylated Tau proteins [5, 6], and the loss of neurons [7]. The prevailing “amyloid cascade hypothesis” indicates that A*β* aggregation plays a critical role in the pathogenesis of AD [8–10]. Though the mechanisms underlying A*β*-mediated neurotoxicity still remain elusive, heat shock proteins are recognized as major contributors [11, 12].

Heat shock proteins (Hsps) are a class of molecular chaperons facilitating the folding of other proteins to ensure their maintenance of native conformation under stress or other toxic conditions [12, 13]. Heat shock protein 70 (Hsp70) is a major member of Hsps family and plays an important role in a complex neuroprotective system [11, 14]. Virally mediated Hsp70 overexpression rescued neurons from the toxic effects of intracellular A*β* accumulation [15], and exogenous Hsp70 can reduce A*β* plaque formation in 5XFAD mice [16]. Yurinskaya and his coworkers demonstrated that the effect of Hsp70 is realized via reduction of the oxidative stress and apoptosis induced by the peptide isoAsp7-A*β*(1–42) in human neuroblastoma cells [17]. Glucose-regulated protein 78 (Grp78) is the solo endoplasmic reticulum (ER) homologue of Hsp70 and maintains the homeostasis of ER via participating in the process of protein folding and assembly and translocation of protein across the ER membrane. ER is an organelle coordinating synthesis, folding, exporting, and degradation of proteins, and evidence shows that endoplasmic reticulum stress (ERS) is closely related to AD [18, 19]. When misfolded proteins, like A*β*, accumulate in ER, Grp78 releases and activates ERS to restore the homeostasis of ER. However, when stress is prolonged or severe, cell apoptosis happens. Hsp70 can not only act as molecular chaperon helping proteins refold, but also enhance cells' ability to resist damage caused by oxidative stress [11, 15]. Moreover, evidence shows that A*β* can induce ERS and activate ER-related and mitochondria-related apoptosis [20, 21], so the problem here is to elucidate the role that Hsp70 and Grp78 play in A*β*-induced ERS and apoptosis.

Herbs have been widely used for thousands of years in China and with little serious side effects. Furthermore, compared with single-component drugs, Traditional Chinese Medicine (TCM) drugs exhibit a multicomponent, multipathway, and multitargets advantage and are able to treat multifactor, complex chronic diseases such as AD. Yi-Zhi-Fang-Dai formula (YZFDF), which is prescribed on the basis of clinical experience, is commonly used in clinic of TCM to treat dementia. YZFDF is composed of several compounds, including bilobalide, ginkgolide A, ginsenoside Rb1, ginsenoside Rg1, cistanoside A, and *α*-asarone. Our previous studies show that the extract of* Ginkgo biloba* leaves (EGb761), the main herb of YZFD formula, can protect against A*β*-induced cell damage [22, 23]. In a previous study, it has been reported that ginsenosides can restore metabolite imbalance in AD mice [24]. Wu et al. [25] suggested that* Cistanche tubulosa* extract could ameliorate the cognitive dysfunction in AD-like rat model. Moreover, *α*-asarone can inhibit proinflammatory cytokines and microglial activation in the hippocampus and ameliorate memory deficits [26].

This study aimed to examine the potential neuronal protective effect of YZFD formula against A*β*-induced neurotoxicity in SH-SY5Y cells. Our previous study indicates that A*β*_1–42_ oligomer showed more efficient neurotoxicity in SH-SY5Y cells than a scrambled A*β*_42–1_ peptide and 10 *μ*M A*β*_1–42_ had a significant neurotoxicity effect on SH-SY5Y cells [22]; thus, we used 10 *μ*M A*β*_1–42_ oligomer to treat cells mimicking AD cell damage model in this study. We examined the effects of YZFD formula on reducing A*β*-induced neurotoxicity through increasing the expression of Hsp70 and Grp78. In this study, our data support the possibility that YZFD formula might have protective effects, including the attenuation of neuron cell apoptosis and associated molecular chaperones.

## 2. Materials and Methods

### 2.1. Regents and Antibodies

Lyophilized human A*β*_1–42_ purified by HPLC was purchased from GL Biochem (Shanghai, China). We bought the rabbit anti-Hsp70, anti-Grp78, anti-caspase-12, anti-caspase-3, anti-p-Akt, and mouse anti-*β*-actin from Cell Signaling Technology (MA, USA) and rabbit anti-Akt1 from Millipore (MA, USA). 3-(4,5-Dimethylthiazol-2-yl)-2,5-diphenyltetrazolium bromide (MTT) was purchased from Sigma (CA, USA), and annexin V-FITC/PI Apoptosis Detection Kit was purchased from Beyotime (Shanghai, China).

### 2.2. Preparation of YZFDF Drug Powder

Four herbs were used in this study, including* Ginkgo biloba* leaves, ginseng, Cistanches Herba, and grass leaved sweetflag. All these herbs were purchased from Shanghai Hongqiao Pharmaceutical Co., Ltd. (Shanghai, China) and identified by TCM Preparation Room of Shanghai Geriatric Institute of Chinese Medicine, Shanghai University of Traditional Chinese Medicine. The main effective compounds of YZFDF were identified by Shanghai Jiao Tong University School of Pharmacy. The extracts of YZFDF were obtained as follows: 500 g of four herbs was subjected twice to extraction with 75% ethanol for 2 h. The dregs of the decoction were removed after filtering. The filtered liquid was concentrated by Rotary Evaporator (BÜCHI Labortechnik AG, Flawil, Switzerland) and then dried using freeze drying method to get drug powder of 158.6 g. The YZFDF drug powder were stored at 4°C and dissolved in DMSO at a concentration of 200 mg/mL and then the required concentrations of YZFDF were prepared from the 200 mg/mL solution diluted in Dulbecco's modified Eagle's medium (DMEM, Gibco, USA), a cell culture medium.

### 2.3. Identification of the Extracts of YZFDF

**Figure 1:**
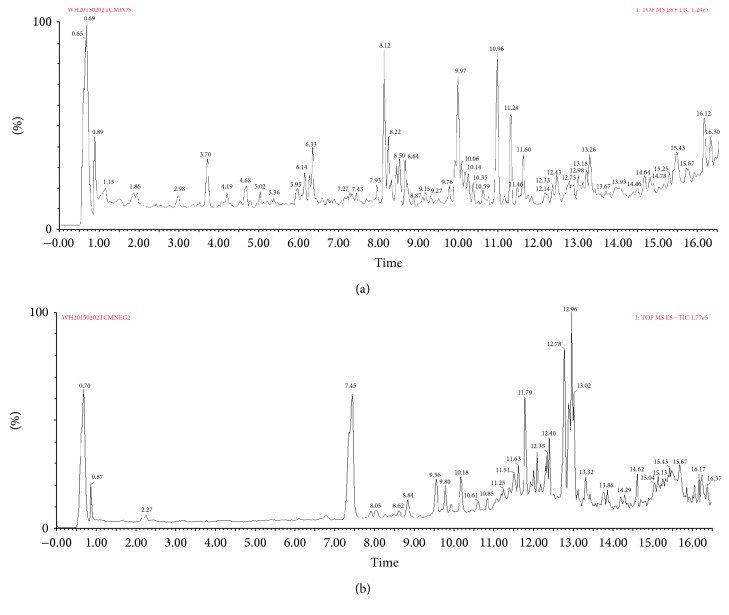
High-performance liquid chromatography (HPLC) analysis of Yi-Zhi-Fang-Dai formula (YZFDF). (a) The HPLC analysis spectrum of YZFDF in positive ion mode. (b) The HPLC analysis spectrum of YZFDF in negative ion mode.

**Figure 2:**
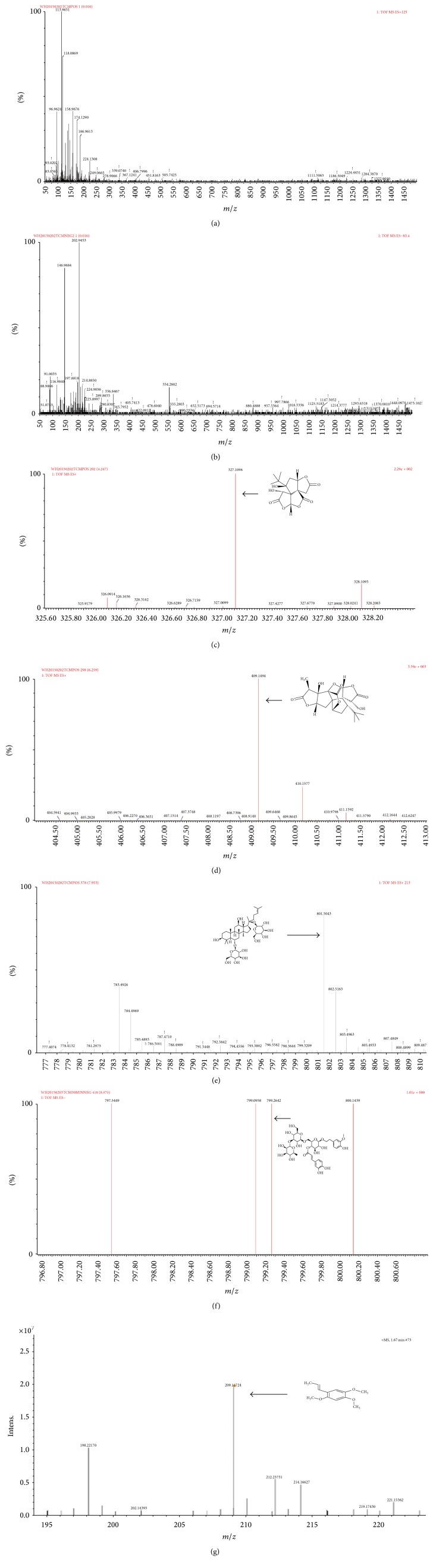
Chemical structures and mass spectrometry (MS) analysis spectrum of YZFDF. (a) The MS analysis spectrum of YZFDF in positive ion mode. (b) The MS analysis spectrum of YZFDF in negative ion mode. (c) The chemical structure and [M + H]^+^ MS analysis spectrum of bilobalide. (d) The chemical structure and [M + H]^+^ MS analysis spectrum of ginkgolide A. (e) The chemical structure and [M + H]^+^ MS analysis spectrum of ginsenoside Rg1. (f) The chemical structure and [M + H]^–^ MS analysis spectrum of cistanoside A. (g) The chemical structure and [M + H]^+^ MS analysis spectrum of *α*-asarone.

An Agilent 1100 HPLC system (Santa Clara, CA, USA) coupled with Fourier Transform Ion Cyclotron Resonance solariX 7.0T (Bruker Daltonics Inc., USA) and High Liquid Chromatography & Linear Ion Trap Quadrupole LTQ XL (Thermo Scientific, San Jose, CA, USA) were used for analysis of the extracts of YZFDF. Samples were prepared as follows: 3 g YZFDF drug powder was dissolved in 100 mL 70% ethanol with ultrasonic and then the extracted solution was dried with Rotary Evaporator. Stock solutions of main components of YZFDF were prepared in methanol and stored at 4°C. Working solutions were prepared on the day of analysis by further dilution of the stock solutions with methanol. The parameters for HPLC were as follows: for HPLC system coupled with solariX 7.0 T, chromatographic separation was performed on an Agilent ODS C18 column (50 mm × 2.0 mm, 5 *μ*m) at room temperature and using a mobile phase that consisted of methanol-1% acetic aqueous solution (74 : 26) at a flow rate of 0.3 mL/min; for HPLC system coupled with LTQ XL, chromatographic separation was performed on an Agilent ODS C18 column (250 mm × 4.6 mm, 5 *μ*m) at 35°C and the mobile phase was methanol in distilled deionized water (60/40, v/v) at a flow rate of 1.0 mL/min. The MS was operated with electrospray ionization (ESI) interface in positive and negative ionization mode for YZFDF. The ionization source conditions were capillary voltage 3.0 kV, cone voltage 55 V, source temperature 100°C, and desolvation temperature 250°C. The data was collected between 50 and 1000 *m*/*z* with the optimized collision energy at 6.0 V for YZFDF. The cone and desolvation gas flow rates were 50 and 600 L/h, respectively. The HPLC analysis spectrum is shown in Figure 1, while the chemical structures of each component and MS analysis spectrum are shown in Figure 2.

### 2.4. Cell Culture and Treatments

Human neuroblastoma SH-SY5Y cells were grown and maintained in DMEM supplemented with glucose (4.5 g/L), fetal bovine serum (10%, Gibco, USA), penicillin (100 U/mL), and streptomycin (100 *μ*g/mL) at 37°C with 5% CO_2_. The cells were pretreated with various concentrations of YZFDF for 2 h and then treated with 10 *μ*M A*β*_1–42_ oligomer for 24 h. The 10 *μ*M A*β*_1–42_ oligomers were prepared as mentioned previously [22]. In brief, 1 mg A*β*_1–42_ was initially diluted in 220 *μ*L ice-cold hexafluoroisopropanol (HFIP, Sigma, USA) and then HFIP was removed under vacuum in a Speed Vac, and the peptide was stored at −20°C. For oligomer preparation, 2 mM A*β*_1–42_ dissolved in DMSO was diluted in ice-cold Opti-MEM (Gibco, USA) to bring the peptide to a final concentration of 100 *μ*M and then incubated at 4°C for 24 h before use.

### 2.5. Cell Viability Assays

After various treatments, the viability of cells was determined by the MTT assay. In brief, cells were seeded on 96-well culture plates overnight, and then 20 *μ*L MTT (5 mg/mL) was added to each well for 4 h at 37°C. 100 *μ*L DMSO was added to solubilize the colored formazan product after the medium was aspirated. At last, the OD value of each well was detected at 490 nm using a microplate reader (BioTek, VT, USA). Cell viability (%) was expressed as a percentage relative to the untreated control cells.

### 2.6. Western Blotting

Following treatment, cells in each 6 cm culture plate were collected and the protein concentration was measured using the Bradford method. Equal amounts of protein were denatured and separated with 10% sodium dodecyl sulfate-polyacrylamide gel electrophoresis (SDS-PAGE). Proteins were then transferred to nitrocellulose membranes (Millipore, USA) and were blocked with blocking buffer (Beyotime) for 1 h at room temperature. The membrane was incubated with primary antibodies (1 *μ*g/mL) overnight at 4°C, followed by incubation with secondary antibody (1 *μ*g/mL) (LI-COR, USA) at room temperature. Images were captured by the Odyssey infrared fluorescence imaging system (LI-COR, USA).

### 2.7. Quantitative Real-Time PCR (qRT-PCR)

qRT-PCR assays were performed with the real-time PCR detection system (Eppendorf) using total RNA and the ReverTra Ace qPCR RT Kit with SYBR green (TOYOBO, Japan). Sequences of the upstream and downstream PCR primers to detect human Hsp70 mRNA used in qRT-PCR were 5′-GCC ACT CTG CTT ATC AAG TTT C-3′ and 5′-CTC CCA ATG TCG TGT CAA AT-3′, respectively. Upstream and downstream primers for human Grp78 mRNA were 5′-AAA GAA ACC GCT GAG GCT TAT-3′ and 5′-CTG AAA CAG TAT GCC GAC AAG-3′, respectively. Upstream and downstream primers for human 18S rRNA were 5′-CAG CCA CCC GAG ATT GAG CA-3′ and 5′-TAG TAG CGA CGG GCG GTG TG-3′, respectively.

### 2.8. Statistical Analysis

All data was expressed as the mean ± SEM. Statistical analysis was performed using IBM SPSS Statistics 19. One-way analysis of variance (ANOVA) followed by LSD (Least Significant Difference) test was used to compare the means of three or more normally distributed samples. Student's *t*-test was used for the evaluation of differences between two groups. Differences were considered to be significant for values of *p* < 0.05.

## 3. Results

### 3.1. Structure Identification of Chemical Compounds of the Extracts of YZFDF by HPLC-MC

Most of the main effective compounds of extracts of YZFDF are bilobalides and ginsenosides which exhibited their protonated-molecular ions [M − H]^+^ in positive ion mode, while the main compounds of Cistanches Herba exhibited their deprotonated-molecular ions [M + H]^–^ in negative ion mode. As shown in Figure 2, we identified bilobalide and ginkgolide A, the main compounds of* Ginkgo biloba* leaves, and ginsenoside Rg1, the main compound of ginseng, and cistanoside A of Cistanches Herba in the extracts of YZFDF. *α*-Asarone is a kind of volatile small molecule, which can be detected by using atmospheric pressure chemical ionization (APCI), and the results showed that the main compound of grass leaved sweetflag, *α*-asarone, existed in this extract of YZFDF.

### 3.2. YZFDF Can Increase Cell Viability against A*β*_1–42_ Oligomer's Toxicity

**Figure 3:**
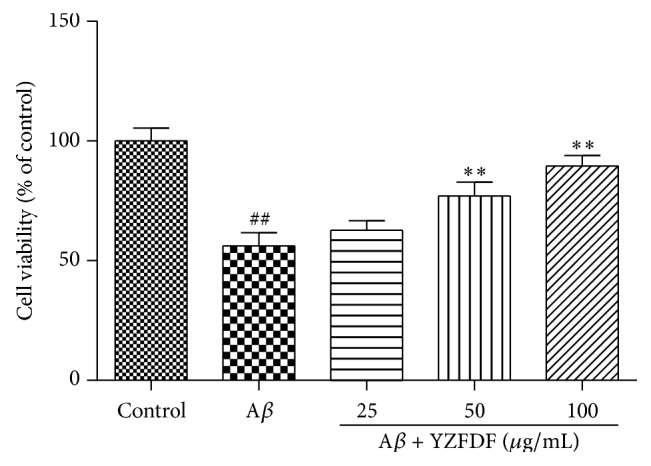
YZFDF increased SH-SY5Y cell viability against A*β*_1–42_ oligomer toxicity. Cells were pretreated with or without various concentrations of YZFDF for 2 h and then incubated with 10 *μ*M A*β*_1–42_ oligomer for 24 h. Subsequently, cell viability was measured by the MTT assay. The results are shown as mean ± SEM (^##^ *p* < 0.01, control versus A*β*; ^*∗∗*^ *p* < 0.01, A*β* + YZFDF versus A*β*).

To investigate the effects of YZFDF on A*β*_1–42_ oligomer induced neurotoxicity, SH-SY5Y cells were pretreated with or without YZFDF for 2 h and then incubated with 10 *μ*M A*β*_1–42_ oligomer for 24 h. As shown in Figure 3, we can find that cells treated with A*β*_1–42_ oligomer alone showed significant low viability compared to cells in the control group. Furthermore, in cells pretreated with various concentrations of YZFDF, cell viability increased in a dose-dependent manner. Besides, in cells pretreated with 50 *μ*g/mL and 100 *μ*g/mL YZFDF, cell viability was significantly increased in contrast to cells treated with A*β*_1–42_ oligomer alone.

### 3.3. YZFDF Protects SH-SY5Y Cells from A*β*_1–42_ Oligomer Induced ER-Related Apoptosis

**Figure 4:**
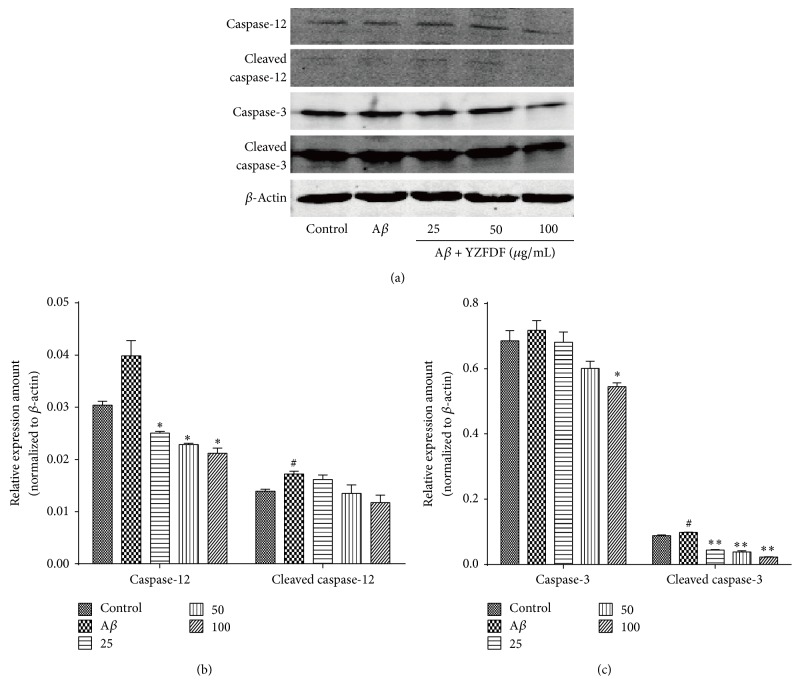
YZFDF protects SH-SY5Y cells from A*β*_1–42_ oligomer induced ER-related apoptosis. YZFDF decreased the expression of caspase-12, caspase-3, cleaved caspase-12, and cleaved caspase-3 in A*β*_1–42_ oligomer treated cells in a dose-dependent manner. The results are shown as mean ± SEM (^#^ *p* < 0.05, control versus A*β*; ^*∗*^ *p* < 0.05, ^*∗∗*^ *p* < 0.01, A*β* + YZFDF versus A*β*).

Caspase-12 is a member of apoptosis protein family produced by ER. The malfunction of ER leads to the activation ERS, with a subsequent increased expression of cleaved caspase-12, with the activation of ER-related cell apoptosis in the end. As shown in Figure 4, in cells pretreated with various concentrations of YZFDF, the expression of caspase-12 and caspase-3 decreased in a dose-dependent manner while A*β* treated alone group showed an increase in expression of caspase-12 and caspase-3. Evidence of caspases activation is provided by the proteolysis of procaspases into smaller cleaved caspases fragments. Thus, we detected the expression of cleaved caspase-12 and caspase-3. Compared to control group, A*β* treated alone group expressed an increased expression of cleaved caspase-12 and cleaved caspase-3, while YZFDF pretreatment restored the increased expression of cleaved caspase-12 and cleaved caspase-3 dose-dependently, especially cleaved caspase-3.

### 3.4. YZFDF Increased the Expression of Hsp70 and Grp78 in SH-SY5Y Cells

**Figure 5:**
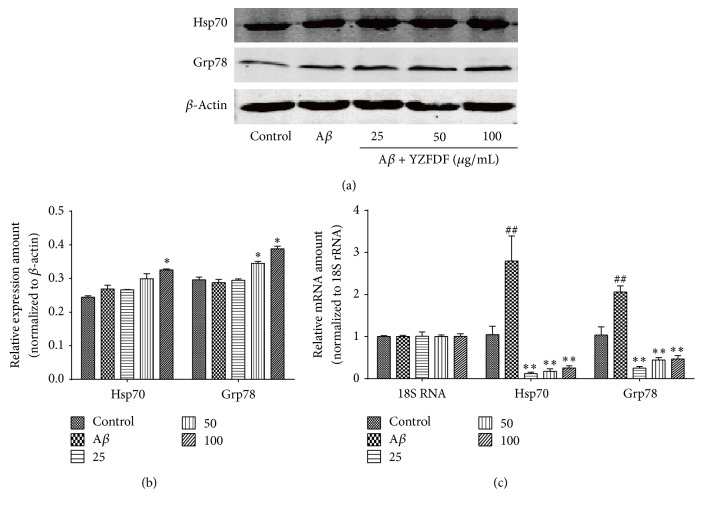
YZFDF influenced the protein and mRNA expression of Hsp70 and Grp78 in SH-SY5Y cells. Cells were pretreated with or without various concentrations of YZFDF for 2 h and then incubated with 10 *μ*M A*β*_1–42_ oligomer for 24 h. (a) and (b) showed that YZFDF increased Hsp70 and Grp78 expression in SH-SY5Y cells in a dose-dependent manner compared to that seen for treatment with A*β*_1–42_ oligomer alone. (c) showed that YZFDF could increase Hsp70 and Grp78 mRNA expression in a dose-dependent manner, while the mRNA expression was lower than that seen for treatment with A*β*_1–42_ oligomer alone. The results are shown as mean ± SEM (^##^ *p* < 0.01, control versus A*β*; ^*∗*^ *p* < 0.05, ^*∗∗*^ *p* < 0.01, A*β* + YZFDF versus A*β*).

To explore the relationship between ER stress and the neuroprotective effect of YZFDF, we further investigate the effect of YZFDF on the expression of Grp78 (an ER stress marker) and Hsp70. The data showed that, in cells pretreated with YZFDF, the protein expression of Grp78 and Hsp70 is significantly higher than in cells treated with A*β* alone in a dose-dependent manner (Figures 5(a) and 5(b)). However, the PCR results showed that A*β* treated alone group manifested a significant higher mRNA expression of Hsp70 and Grp78 than other groups, while YZFDF pretreated groups still showed a dose-dependent increased mRNA expression of Hsp70 and Grp78 (Figure 5(c)).

### 3.5. YZFDF Can Activate the Akt in SH-SY5Y Cells

**Figure 6:**
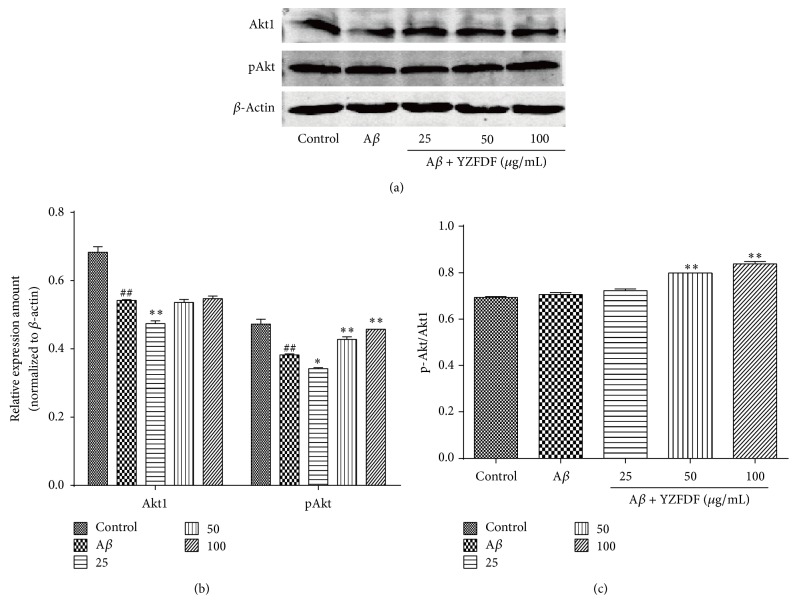
YZFDF activated the Akt expression in a dose-dependent manner. Cells were pretreated with or without various concentrations of YZFDF for 2 h and then incubated with 10 *μ*M A*β*_1–42_ oligomer for 24 h. The data are shown as mean ± SEM (^##^ *p* < 0.01, control versus A*β*; ^*∗*^ *p* < 0.05, ^*∗∗*^ *p* < 0.01, A*β* + YZFDF versus A*β*).

Evidence suggested that Akt pathway is a prosurvival signaling system in neurons [27]. To identify whether the neuroprotective effect of YZFDF is related to the activation of Akt, we further used the western blotting method to explore the expression of Akt1 and pAkt. As shown in Figure 6, we can see that A*β*_1–42_ oligomer decreased the expression of Akt1 and pAkt, while, in cells pretreated with YZFDF, Akt1 and pAkt protein expression increased dose-dependently (Figure 6(b)). Besides, compared to A*β* treated alone group, 50 *μ*g/mL and 100 *μ*g/mL YZFDF pretreated groups showed significant Akt activation (Figure 6(c)).

## 4. Discussion

In this study, the effective compounds of YZFDF have been identified. We identified the main compounds of* Ginkgo biloba* leaves, bilobalide and ginkgolide A, and found ginsenoside Rg1, the main compound of ginseng, in YZFDF samples. Moreover, we also identified cistanoside A of Cistanches Herba and *α*-asarone of grass leaved sweetflag in YZFDF samples. Our previous studies found that the EGb761, the extract of* Ginkgo biloba* leaves which is an important herb in this formula, exhibited a good protective effect on endothelial cells [23] and neuron cells [22]. In this work, the integral formula also restored SH-SY5Y cell viability from A*β*_1–42_ oligomer induced decreased cell viability (Figure 3).

The “amyloid cascade hypothesis” indicates that the aggregation of A*β* triggers a series of downstream events including the formation of neuritic plaques and neurofibrillary tangles and neuronal loss and ultimately clinical dementia [28, 29]. Available data presents abnormal accumulation of A*β* as a key factor, which can result in mitochondrial and ER dysfunction and eventually cell apoptosis [30]. Caspases have a central role in mammalian cell apoptosis and are classified into two different groups, namely, initiator and effecter caspases. Caspase-12 is an ER-specific initiator caspase, which, later on, activates the effector caspase-3, eventually leading to ER-mediated cell death pathway. Our data showed that YZFDF can not only decrease the expression of procaspase-12 and procaspase-3, but also decrease the expression of activated caspases, cleaved caspase-12, and cleaved caspase-3, while, in A*β*_1–42_ treated alone group, the expression of these apoptosis proteins increased (Figure 4). This indicates that YZFDF can increase neuron cell viability via attenuating ER-related apoptosis and A*β*_1–42_ can induce ER-related apoptosis in SH-SY5Y cells.

Evidence showed that exogenous A*β* could induce ERS and activate mitochondria- and ER-mediated cell apoptosis [31, 32]. As a result of ERS, Grp78, the marker protein of ERS, dissociates from ER domains and binds to overloaded A*β*, to reestablish homeostasis [33], and Hsp70, the homologue of Grp78, plays a key role in maintaining protein homeostasis via participating in helping protein refolding; thus, we further explored the expression of Hsp70 and Grp78. Our data showed that A*β*_1–42_ can significantly increase the mRNA expression of Hsp70 and Grp78, but the protein expression of Hsp70 and Grp78 did not show a significant increase (Figure 5). On the other hand, the western blotting results showed that YZFDF increased the expression of Hsp70 and Grp78 in SH-SY5Y cells compared to A*β* treated alone group, while the PCR results showed a lower expression, but a dose-dependent increased expression in YZFDF pretreated groups (Figure 5). The lower expression of mRNA and the higher expression of protein of YZFDF pretreated groups compared to A*β* treated alone group gave us a clue that YZFDF can influence ERS and protein folding process to play a role in neuroprotection, but the precise mechanism about the relationship between lower mRNA expression and higher protein expression needs further exploration. What is more, we detected whether the attenuation of ER-mediated apoptosis caused by YZFDF pretreatment had a relationship with Akt activation. Our results showed that YZFDF pretreatment could increase the expression of Akt1 protein and pAkt protein, inducing activation of Akt in a dose-dependent manner, while A*β*_1–42_ treated alone group showed a decreased protein expression of Akt1 and p-Akt (Figure 6); this may reveal a relationship between Akt activation and ER-related apoptosis. All these data showed that YZFDF had a strong neuroprotective effect against A*β* neurotoxicity.

Nowadays, there still exist little effective therapeutic methods to cure AD. With the advantages of multitargets, multipathways, and multicomponents, TCM herbs show a good curative effect with little side effects and should be a candidate for curing neurodegenerative diseases like AD. Our experiential prescription, YZFDF, has shown a strong neuroprotective effect against A*β* through attenuating ER-mediated apoptosis, mediating protein folding process and ERS, and activating Akt expression. This study highlights the potential for YZFDF to be a potent drug candidate for the treatment of AD. However, further studies on this formula like the precise roles of its effective compounds in neuroprotection and the clear mechanism of its neuroprotective effect in restoring the AD pathology still need to be done in the future.
